# CASTLE Thyroid Tumor: A Case Report and Literature Review

**DOI:** 10.3389/fonc.2017.00207

**Published:** 2017-09-13

**Authors:** Chris Lominska, Christopher Fleighton Estes, Prakash C. Neupane, Y. Shnayder, Mindi J. TenNapel, Maura F. O’Neil

**Affiliations:** ^1^Radiation Oncology, University of Kansas Medical Center, Kansas City, KS, United States; ^2^Radiation Oncology, Mercy Clinic Radiation Oncology, Springfield, MO, United States; ^3^Medical Oncology, University of Kansas Medical Center, Westwood, KS, United States; ^4^Otolaryngology, University of Kansas Medical Center, Kansas City, KS, United States; ^5^Pathology, University of Kansas Medical Center, Kansas City, KS, United States

**Keywords:** thyroid tumor, CASTLE, case report, thyroid carcinoma, radiation therapy

## Abstract

Carcinoma showing thymus-like differentiation is a rare tumor of the thyroid gland, which is structurally similar to thymic tissue. Overall, it has a favorable prognosis. Radiotherapy has been shown to be an effective local treatment, but there have been reports of distant recurrence. It has been suggested that adding chemotherapy may decrease the risk of recurrence. Here, we present a case report of a patient with a large tumor and extrathyroidal extension. The patient was treated with surgery, radiotherapy, and cisplatin with acceptable toxicity. The patient is free of locally recurrent or distant disease at 3 years.

## Introduction

We report a case of carcinoma showing thymus-like differentiation (CASTLE) and review the literature. The patient has provided their written informed consent for the publication of this manuscript and any identifying images or data. CASTLE is a rare neoplasm of the thyroid gland, with approximately 50 reported cases in the literature to date (1). This entity was first discovered by Miyauchi et al. in 1985 and described as an “intrathyroidal epithelial thymoma” (2). Chan and Rosai in 1991 divided these tumors into four classifications: ectopic hamartomatous thymoma, ectopic cervical thymoma, spindle epithelia tumors with thymic-like differentiation (SETTLE), and carcinoma showing thymic-like elements (CASTLE) (3). SETTLE and CASTLE exhibit malignant characteristics while the other two classifications are considered benign. SETTLE presents in younger age individuals, and CASTLE typically manifests in the fifth decade. Histologically CASTLE shows similarity to thymic tissue. It is suspected to arise from remnants of the thymopharyngeal duct, ectopic thymus, or branchial pouch.

On histopathologic analysis, CASTLE shows structural similarity to thymic tissue. Immunohistochemistry staining shows positivity for CD5 (4) in nearly all, and negativity for markers corresponding to thyroid tissue, such as thyroglobulin and calcitonin (5). On molecular analysis, p63 is overwhelmingly positive in tumors of thymic origin, while negative in follicular carcinoma and poorly differentiated thyroid carcinoma. Papillary thyroid carcinoma shows variable positivity with p63 (5).

Diagnosis with CASTLE typically carries a favorable prognosis. Lymph node metastasis rates are about one-third (5). There are reported cases of progression to metastatic disease (6). In a series of 22 patients, 29% developed recurrence: four distant, two locoregional, and one distant and locoregional. Of the patients in this series, the 5- and 10-year CSS rates were 90 and 82%, respectively (7).

## Case Report

A 60-year-old gentleman with past medical history of cervical disk herniation presented with a 3-year history of progressive fatigue and bilateral upper extremity weakness. MRI was performed to evaluate the spine and was consistent with cervical spinal stenosis. T2 non-contrast images incidentally revealed an infiltrating mass in the left thyroid that was incompletely visualized. Ultrasound-guided fine needle aspiration showed atypia of undetermined significance. The patient was referred to surgery and was noted to have dysphonia but no stridor or dysphagia. The differential diagnosis was felt to include papillary thyroid carcinoma, follicular thyroid carcinoma, rare thyroid neoplasms including Hurthle cell carcinoma or anaplastic carcinoma, abscess, or autoimmune thyroid disease. He underwent total thyroidectomy with central neck dissection. On exploration, the thyroid mass was seen encasing the left recurrent laryngeal nerve and was shaved off the nerve and the trachea. The nerve was not stimulatable after resection and the patient developed left vocal cord paralysis. The tumor grossly measured 7 cm with extrathyroidal extension. There was lymphvascular space invasion and perineural invasion. Three lymph nodes were identified, and all were negative. Surgical margins had extensive involvement of tumor. Histopathologic analysis of the specimen showed high-grade carcinoma with thymus-like differentiation. Immunohistochemical staining was positive for pan-CK, p63 (cyokeratin and epithelial markers) and negative for chromogranin, thyroglobulin, and calcitonin (neuroendocrine, papillary and follicular thyroid, and medullary makers, respectively).

**Figure 1:**
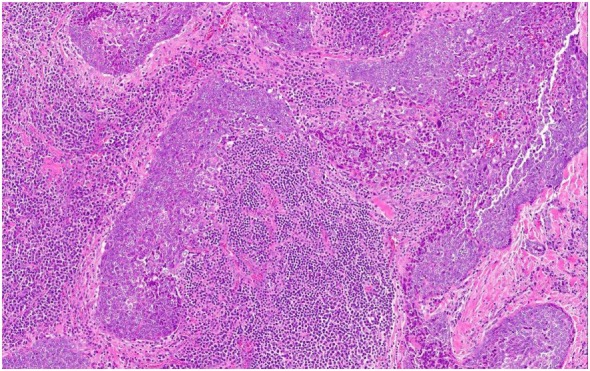
The tumor cells are arranged in broad, pushing, smooth-bordered islands abutted against a lymphocyte rich stroma and seen in 10× (Figure 1) and 20× (Figure 2).

**Figure 2:**
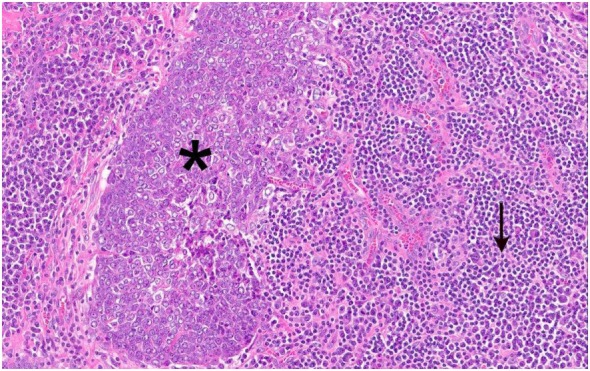
Figure 1 magnification. The asterisks highlights the lobules of tumor in the images. These are surrounded by lymphocytes and plasma cells, as indicated by the arrow.

**Figure 3:**
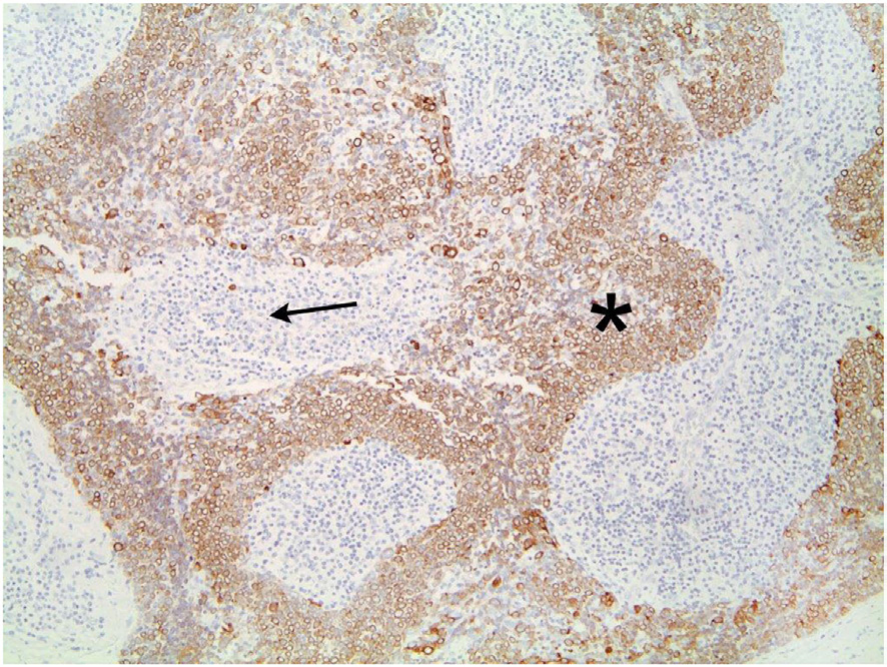
The neoplastic cells are strongly immunoreactive for pancytokeratin. Lymphocytes are negative.

**Figure 4:**
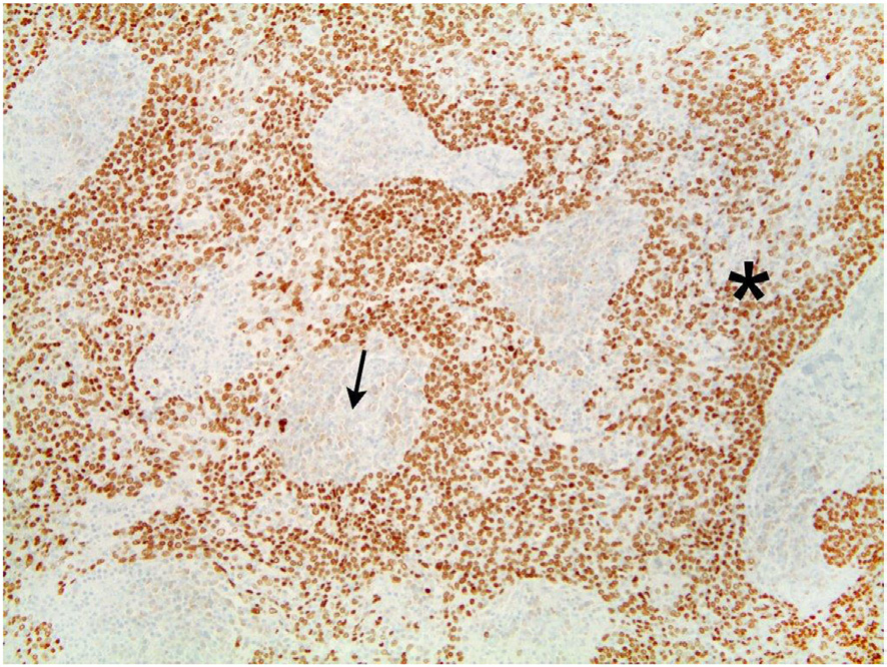
The tumor cell shows strong immunoreactivity to p63. Lymphocytes lack reactivity.

**Figure 5:**
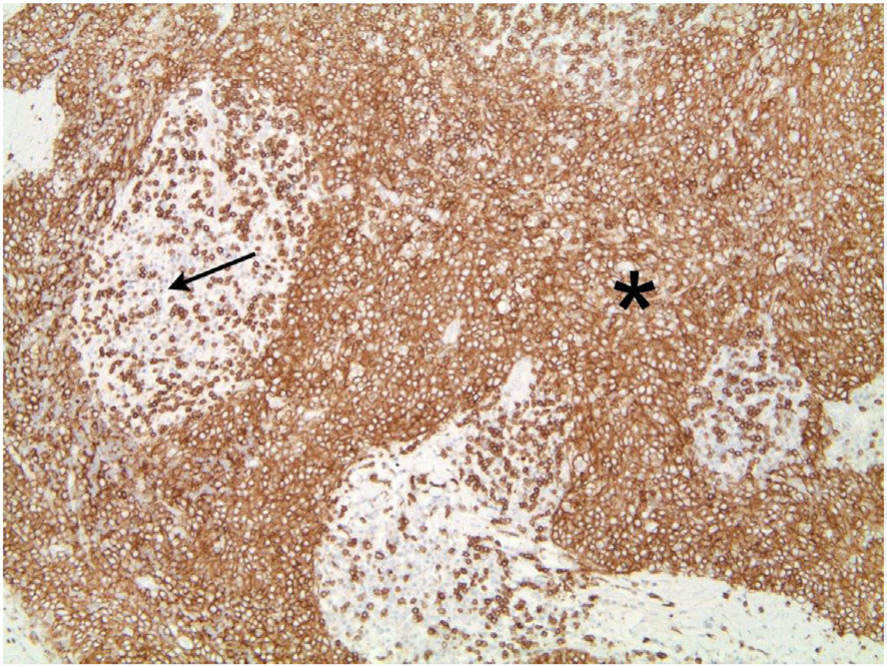
Tumors cells that demonstrate strong positivity to CD5, also marking background lymphocytes.

The tumor was arranged in broad, pushing, smooth-bordered islands abutted against a lymphocyte rich stroma. The lobules of tumor were surrounded by lymphocytes and plasma cells (Figures 1 and 2). The tumor cells were squamoid and syncytial to spindled, with eosinophilic cytoplasm, and the nuclei show mild pleomorphism with pale vesicular chromatin. The neoplastic cells were strongly immunoreactive for pancytokeratin and p63, as shown in Figures 3 and 4. These cells were also strongly positive for CD5, which marks background T lymphocytes (Figure 5). The neoplastic cells were non-reactive with TTF1 and thyroglobulin.

**Figure 6:**
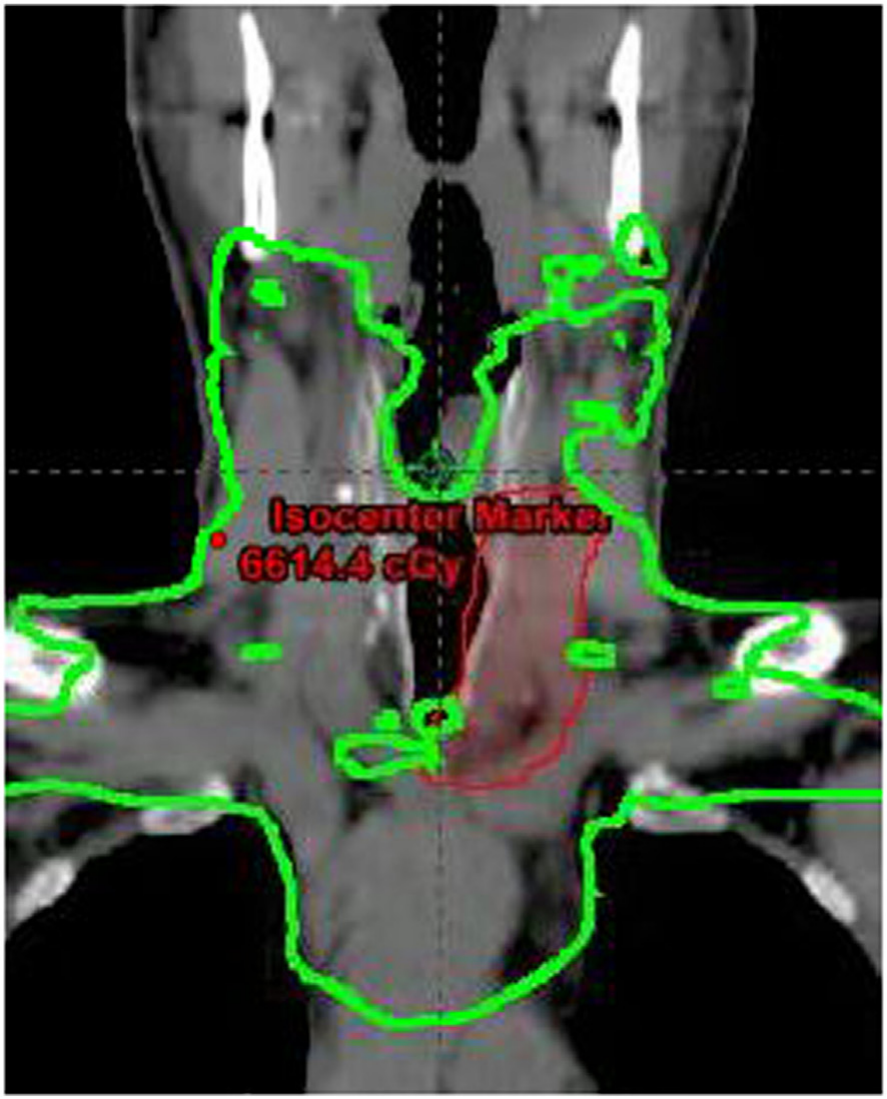
Coronal section of radiotherapy plan showing 60 Gy dose line in green and boost volume in red.

Postoperative staging surveillance with PET scan was performed for systemic staging and due to positive margins. It showed no abnormal FDG uptake at distant sites and increased uptake at the operative bed, indeterminate between residual disease and postoperative changes. The patient underwent adjuvant treatment with radiation therapy to 64 Gy in 32 fractions with intensity modulated radiotherapy. Concurrent chemotherapy was given based on his extensive positive margins for radiosensitization. A dose of 60 Gy was delivered to the bilateral neck including paratracheal nodes into the upper mediastinum, with a 4 Gy sequential boost to the operative site. A coronal section of the radiotherapy plan is shown in Figure 6. Six cycles of intravenous concurrent cisplatin 35 mg/m^2^ were infused. During therapy, he developed grade 2 skin reaction characterized by dry desquamation and moderate erythema of the neck and upper chest. Skin moisturizer was used for therapy. He experienced grade 2 esophagitis and mucositis of the oropharynx and grade 2 nausea requiring topical analgesics, oral narcotics, antiemetics, and intravenous fluid administration. Weight decreased from 108 to 95.5 kg by the end of therapy. Nutritional counseling by dietician and oral nutritional supplementation were provided. With chemotherapy, the patient developed a tinnitus grade 1. Toxicities are reported per the Common Terminology Criteria for Adverse Events (8).

After treatment, the patient initially experienced continued weight loss, and as a result underwent percutaneous endoscopic gastrostomy tube placement. Five months after end of treatment, the tube was removed after the patient was able to gain significant weight and completely obtain nutrition per oral route for 6 weeks. Dysphagia to solid foods is improving. He experienced persistent dysphonia secondary to left vocal cord paralysis since surgery and underwent left medialization thyroplasty 1 year later. He displayed improvement in voice quality. At 3-years follow-up after completion of radiotherapy, our patient is without evidence of locally recurrent or distant disease.

## Discussion

Radiotherapy has demonstrated efficacy in the treatment of CASTLE tumors, primarily with the presence of lymph node involvement, gross disease, or recurrence in case series and reports. In a review of 117 tumors of the thymus, Curran et al. (9) showed total resection alone appears to yield an unacceptably high failure rate, and the addition of radiotherapy can provide high rates of local control. Of these patients, those with tumor extension to adjacent organs were treated with adjuvant radiotherapy. Four out of 10 patients treated with radiotherapy experienced a recurrence compared to 27% for those who did not, likely reflecting the already worse prognosis of those who received radiation. All recurrences were distant from the radiation field.

Chow et al. (10) described three patients with gross disease treated with radiation therapy. On workup, these patients were found to have advanced T4 disease with extensive tracheal infiltration and airway compression. None of these patients were able to undergo complete resection: two had debulking of the tumor, while one was initially inoperable. In the inoperable individual, treatment was initiated with neoadjuvant carboplatin and etoposide followed by radiation therapy to 66 Gy. After successful reduction in size of tumor, total thyroidectomy was performed. On microscopic analysis, residual tumor islands were noted, the largest measuring 3 mm. All three patients received radiotherapy and were free of recurrence with 1.8–6 years of follow-up, demonstrating the utility of radiation therapy in the treatment of gross disease of CASTLE histology.

A series by Tsutsui et al. (1) recommends radiation therapy when patients are found to have lymph node metastases. They report a patient who underwent debulking surgery due to extent of tumor. This patient underwent adjuvant external beam radiotherapy and subsequently was found to have complete response on bronchoscopy. Several years later, the patient developed pulmonary metastases and ultimately died of disease at 10 years. In these patients, the risk of death due to distant disease in the presence of local control raises consideration for using chemotherapy to lower the risk of distant recurrence.

Roka reports a series of 22 patients (11). Of the 15 patients with involved or unknown lymph node status, 5 were treated with surgery and radiotherapy with only one recurrence (20%), whereas 10 were treated with surgery alone and seven recurred (70%). In addition, one patient with one local and two distant recurrences was successfully treated with radiation and chemotherapy. They noted that radiotherapy seems indicated with lymphadenopathy, and that it can be effective for recurrence. In a review by Sun et al. (12), five patients with CASTLE, treated with definitive surgery and radiation therapy to a median dose of 56 Gy, were found to be without tumor recurrence at median follow-up of 34 months. They also reported experience with two patients with local recurrences who are disease free: one underwent resection of the recurrence followed by treatment with adjuvant radiotherapy, and the second underwent complete resection of the recurrence followed by chemotherapy. Radiotherapy has also been employed to treat distant lesions. One patient treated with surgery alone, developed a thoracic spine metastasis, which was successfully treated with radiotherapy with no recurrence identified at 18-month follow-up (13). A Korean case review reports that the three CASTLE patients treated with radiotherapy in their series have shown no recurrences at 49 months of follow-up (14).

In our patient with a large tumor and extrathyroidal extension, we employed radiotherapy to treat microscopic disease for local control. Chemotherapy was added for radiosensitization due to extensive positive margins, extrapolating from the benefit seen in head and neck squamous cell carcinomas with positive margins (15). A potential local control benefit for concurrent chemoradiation (primarily Adriamycin) for non-anaplastic thyroid carcinoma was reported by authors from Memorial Sloan Kettering Cancer Center. The issue of positive margins is not specifically addressed in the reported literature (16). Dose of 63–66 Gy is typically used in the setting of positive margins. Given the proximity of dose limiting structures in the low neck such as the trachea and esophagus, we opted to treat in the adjuvant setting rather then watch and treat in the event of recurrence. We are unable to specifically comment on the utility of PET in this disease, but it is part of our institutional practice in staging for locally advanced, non-iodine avid thyroid neoplasms. We report a patient successfully treated with combined modality therapy of surgery, radiotherapy and cisplatin, with acceptable toxicity.

## Author Contributions

CE and CL composed the manuscript and literature review; MO provided figures and pathology review. CE, CL, MO, PN, MT, and LS had the acquisition, analysis or interpretation of data for the work, revising it critically for important intellectual content, final approval of the version to be published, and agreement to be accountable for all aspects of the work in ensuring that questions related to the accuracy or integrity of any part of the work are appropriately investigated and resolved.

## Conflict of Interest Statement

The authors declare that the research was conducted in the absence of any commercial or financial relationships that could be construed as a potential conflict of interest.
